# Late Presentation of McArdle's Disease Mimicking Polymyalgia Rheumatica: A Case Report and Review of the Literature

**DOI:** 10.1155/crrh/8148736

**Published:** 2025-01-03

**Authors:** Maiar Elghobashy, Ute Pohl, James Bateman

**Affiliations:** ^1^Department of Rheumatology, Royal Wolverhampton NHS Trust, Wolverhampton, UK; ^2^Department of Cellular Pathology, University Hospitals Birmingham NHS Trust, Birmingham, UK; ^3^College of Medical and Dental Sciences, Institute of Clinical Sciences, University of Birmingham, Birmingham, UK

**Keywords:** McArdle, McArdle's, PMR, polymyalgia rheumatica

## Abstract

McArdle disease or glycogen storage disease Type V is a genetic condition caused by PYGM gene mutations leading to exercise intolerance and fatigability. The condition most commonly presents in childhood. In rare cases, patients have presented with late-onset McArdle disease. We present a case of a 64-year-old male presenting with myalgia who was initially presented with polymyalgia rheumatica–type symptoms of proximal muscle pain and a response to steroids. At review, his background musculoskeletal symptoms were evaluated in detail. Following a muscle biopsy, skeletal muscle enzymatic assay, and genetic testing, he was diagnosed with late-onset McArdle's disease (homozygous PYGM genotype). The importance of recognition and early diagnosis is highlighted to enable the accurate diagnosis and conservative lifestyle advice, with the avoidance of other medical therapies for other disease mimics.

## 1. Introduction

McArdle disease, or glycogen storage disease Type V (GSD-V), is an inherited autosomal recessive metabolic disorder, which mainly affects skeletal muscle. It arises as a result of mutations in the muscle-specific version of the glycogen phosphorylase enzyme/myophosphorylase encoded by the PYGM gene located on chromosome 11q13, which is involved in the first stage of glycogenolysis [1, 2]. Therefore, glycogen cannot be converted into glucose 1-phosphate, which prevents the further progression of glycogenolysis to produce glucose [3]. It is estimated that the incidence of McArdle disease is 1 in 100,000, with around 350 people currently diagnosed in the United Kingdom [4].

The disease most commonly presents with exercise intolerance, myalgia, and fatigue. In addition, it may also be associated with sudden painful muscle contractions during exercise, which peak immediately on starting exercise [3]. Symptoms often present in children or young adults; however, several rare cases have been reported to present in older ages. We present a rare case of McArdle disease presenting in the sixth decade of life. The differentials are considered, and an up-to-date review of the literature is discussed.

## 2. Case Presentation

A 64-year-old male initially presented to the rheumatology clinic with severe acute onset myalgia affecting his hips and shoulders. Three months earlier, he had been diagnosed with Type 2 diabetes. He had proximal myalgia and stiffness in the upper limbs more so than the lower limbs, and he explained that the severity of pain was affecting his sleep, without diurnal variation. He had been prescribed morphine for his pain by his primary care doctor. The patient was given a trial of prednisolone 30 mg once daily for 5 days by his general practitioner, which produced a significant improvement in his symptoms. Systemic examination was normal, with no overt neurological deficit, but with nonspecific myalgia and arthralgia.

His past medical history included fatty liver disease and Type 2 diabetes mellitus, and he did not take metformin or atorvastatin. He was a retired company director, nonsmoker, with no significant alcohol intake, and the systems' review was unremarkable. There was no significant family history of note.

Investigations showed raised normal or negative full blood count, PSA, immunoglobulins, renal function, vitamin D, and bone profile. He had chronic mild elevations in alkaline phosphatase and gamma GT. Inflammatory markers were elevated: CRP (peak: 103 mg/mL [< 5]) and ESR (45 mm/Hr). A creatine kinase level was elevated at 811 IU/L (reference range: 30–200 IU/L). Immunological tests including ANA, ENA, and viral serology for hepatitis B and C, HIV, DsDNA, and extended-spectrum myositis autoantibody screen were negative. MRI spine was normal. Upper limb muscle MRI showed no diagnostic findings, with nonspecific atrophy but no edema. A working diagnosis of polymyalgia rheumatica (PMR) was made and he was commenced on a tapering prednisolone regime, initially 20 mg. An EMG was limited due to severe pain but did show some evidence of a possible myopathic disorder that was symmetrical. A muscle biopsy was offered but not performed, as the patient's symptoms had resolved with corticosteroid treatment.

By month 14, the patient was off steroids, but on detailed questioning, he reported different episodic short-lived myalgia, which was normal for him. His CK at the clinic visit, at this point following strenuous activity, clearing a wooded area, had risen to 3473 IU/L, CRP < 5 mg/L. An EMG was repeated, which showed an ongoing active myopathic process, which was symmetrical. There were the EMG findings of short-duration motor unit potentials and fibrillation potentials in the right deltoid and proximal to distal gradient of severity in the upper limbs.

**Figure 1:**
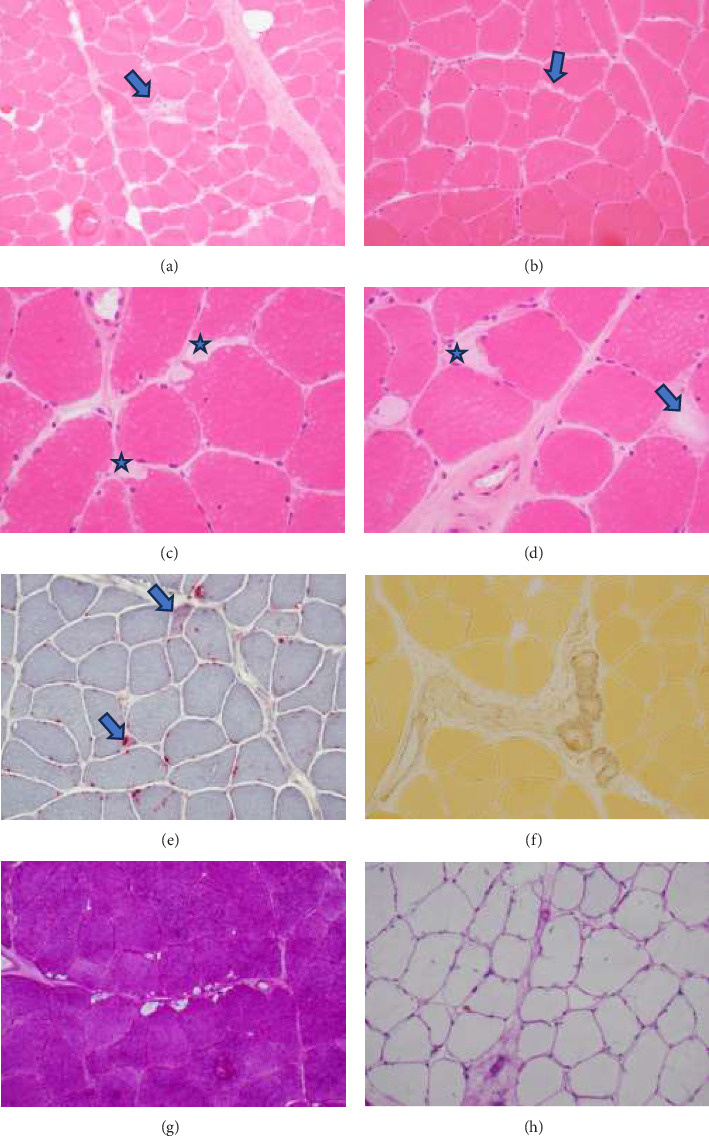
Histological appearances of the muscle biopsy. (a) Occasional necrotic fibers (arrow) were noted surrounded by fibers with increased internalized nuclei (HE × 100 mag.). (b) Some fibers displayed small vacuoles (arrow, HE × 200 mag.). (c and d) Several fibers contain large subsarcolemmal vacuoles (stars, HE × 400 mag.) and occasional pale fibers are seen, consistent with degenerate fibers (arrow in D, HE × 400 mag.). (e) Acid phosphatase was focally positive, compatible with degenerate/necrotic fibers (× 200 mag.). (f) Myophosphorylase was entirely negative in the myofibres (× 200 mag.). (g) PAS histochemistry was strongly positive in sarcoplasm and vacuoles (some PAS-positive material was lost during processing) (× 200 mag.). (h) Treatment with PAS diastase reveals negative staining in sarcoplasm and vacuoles, compatible with glycogen content (× 200 mag.).

A muscle biopsy was then performed. On H&E staining, occasional necrotic fibers were noted with increases in internalized nuclei (Figure 1(a), arrow, HE × 100 mag.). Some fibers displayed small vacuoles (Figure 1(b), arrow, HE × 200 mag), while others harbored large subsarcolemmal vacuoles (Figures 1(c) and 1(d), stars, HE × 400 mag.). Occasional pale fibers were noted, which indicated degenerate fibers (Figure 1(d), arrow, HE × 400 mag.). Scattered split and small angulated fibers were also seen (not illustrated). A histochemical preparation for acid phosphatase was focally positive and compatible with degenerate/necrotic fibers (Figure 1(e)). Histochemistry for myophosphorylase was entirely negative in the myofibres (Figure 1(f)). PAS histochemistry was strongly positive in fibers and vacuoles (although some loss of PAS-positive material during processing occurred), and treatment with PAS diastase revealed negative staining, compatible with glycogen (Figure 1(h)).

Skeletal muscle metabolic analysis (enzymatic assay, Erasmus University Rotterdam, Holland) showed findings consistent with McArdle's with grossly reduced phosphorylase activity (3.9, 75–400 nmol/min/mg) and an elevated muscle glycogen concentration (234.0, 30–180 μg/h/mg). The activities of acid *α*-glucosidase, debranching enzyme, and phosphofructokinase in muscle were normal: no indications for GSD II (M. Pompe), GSD III (M. Cori), or GSD VII (M. Tauri). Further history revealed the classic “second-wind” phenomenon of glycogen storage metabolic myopathy; for example, muscle spasms when walking long distances and having to be carried off a running track following a 50-yard sprint as a child. An exercise diary supported a second-wind phenomenon, with difficulty initially on a rowing machine, which subsided. Further questioning suggested a dietary change following the diagnosis of Type 2 diabetes prior to his presentation could have exacerbated his musculoskeletal symptoms and glycogen deficit. Genetic testing confirmed that he was homozygous for the c.148C > Tp. (arg 50^∗^) pathogenic PYGM gene, confirming McArdle disease.

## 3. Discussion

McArdle disease (GSD-V) is a myopathy secondary to a deficiency in myophosphorylase enzyme due to a mutation in the PYGM gene. Due to its rarity as aforementioned, there may be a delay in the diagnosis of McArdle disease; it has been reported that the frequency of misdiagnosis may be as high as 88% [5]. Although McArdle disease typically presents at a young age, several cases have been reported in the literature of late-onset disease (Table 1). In this case, a deterioration in symptoms from McArdle disease potentially related to the change in diet with a recent diagnosis of Type 2 diabetes, with possible intercurrent, unproven, infection-producing proximal symptoms masquerading as PMR. A response to anti-inflammatory corticosteroids is nonspecific given their potent anti-inflammatory effects. Significant elevation of CK postexercise at follow-up confirms the diagnosis.

There may be several mimics of McArdle disease and differentials which must be considered. First, despite its clinical variation, patients with McArdle disease typically present with symptoms at the start of exercise, whereas other mitochondrial myopathies such as Leigh syndrome and oxidation defects lead to symptoms that appear after prolonged exercise [3]. One defining sign of McArdle disease is the “second-wind phenomenon,” which refers to improvement in exercise tolerance after around 10 min of exercise [11]. Other important features are the persistently elevated CK levels, exercise intolerance, and elevation in CK after exercise [12].

The main differential was PMR due to the age of onset as well as a response to steroids. However, his ongoing symptom after the steroid taper and a persisting CK elevation with a normal CRP led to other diagnostic considerations. PMR is well known to have several disease mimics [13]. Late-onset McArdle disease has itself been reported as mimicking a case of treatment-resistant polymyositis [14]. The definitive method to differentiate McArdle's disease and other conditions is through histology and genetic testing, which was undertaken in this case. Histological findings of McArdle disease include glycogen deposition in the subsarcolemmal areas of the muscle fibers identified through PAS staining. Myophosphorylase staining is low or negative when compared to controls [15].

McArdle disease can be distinguished from other glycogen storage diseases through genetic testing. In recent years, whole exome sequencing has been used in cases to identify pathogenic variants within the McArdle disease spectrum, which may explain the clinical heterogeneity in the presentation of the disease as well as the variability of the age of onset [6]. Nogales-Gadea et al. described that most variants are missense variants with up to 35% of mutations being nonsense variants in the PYGM gene [16]. Further research may be directed in the future in determining whether variants of the PYGM gene mutation affect the age of onset of McArdle's disease.

Treatment of McArdle disease is largely conservative. A large systematic review of RCTs showed that a carbohydrate-rich diet led to improved exercise performance. Similarly, oral sucrose prior to exercise also led to improved exercise tolerance. Drugs such as dantrolene and verapamil did not show any significant benefit [17]. Although McArdle disease is not life-threatening, it does have a significant impact on quality of life, which highlights the importance of an active lifestyle and the aforementioned lifestyle interventions [18]. The main complication of the disease is rhabdomyolysis, which highlights the need for early diagnosis and monitoring for such patients [19].

## 4. Conclusion

McArdle disease is a metabolic myopathy which may mimic more common diseases such as inflammatory myopathies and PMR. This is particularly the case when there is a late onset of symptomology. It is therefore important to consider the differential of McArdle disease in patients with myopathies that are not steroid-responsive and with persistently raised CK levels. Future research may be directed at identifying genetic variants associated with variability in the disease presentation through techniques such as next-generation sequencing and whole exome sequencing.

## Figures and Tables

**Table 1:** Cases of late-onset McArdle disease in patients above 50 in the literature.

Author, year	Age at presentation	Symptoms	CK (IU/L)	EMG	Pathology
Thomas-Wilson et al. 2022 [6]	73	Muscle weakness	Raised, unspecified	—	PYGM gene mutation
Chocair et al. 2020 [7]	81	Muscle weakness, signs of rhabdomyolysis	5141	—	Myophosphorylase staining was negative
Horino et al. 2019 [5]	72	Myalgia and polyarthralgia	4237	—	PYGM gene mutation
Leite, Oliveira, and Rocha 2012 [8]	54	Muscle fatigability on exertion	7924	Myopathic pattern on exertion	Subsarcolemmal accumulation of glycogen
Felice, Schneebaum, and Jones 1992 [9]	60	Mild cramping in upper extremities during exercise, then severe cramps in upper limb	8750	Patchy myopathic process	Scattered fibers with subsarcolemmal collections of PAS-positive material
Pourmand, Sanders, and Corwin 1983 [10]	76	Proximal muscle weakness	—	Spontaneous activity and features of myopathy	Absence of myophosphorylase activity

## Data Availability

All data relevant to the study are included in the article, and further inquiries can be directed to the corresponding author.
